# Transcriptome characteristics during cell wall formation of endosperm cellularization and embryo differentiation in *Arabidopsis*

**DOI:** 10.3389/fpls.2022.998664

**Published:** 2022-10-03

**Authors:** Chengcheng Li, Fan Hu, Hongyu Chen, Jie Zhao

**Affiliations:** State Key Laboratory of Hybrid Rice, College of Life Sciences, Wuhan University, Wuhan, China

**Keywords:** *Arabidopsis*, endosperm cellularization, embryo differentiation, cell wall, transcriptomics, gene expression

## Abstract

Embryonic and endosperm development are important biological events during *Arabidopsis* seed development, and are controlled by dynamic changes in a range of gene expression. Nevertheless, the regulatory mechanisms of endosperm cellularization and embryo differentiation remain unclear. Here, we characterized the early embryo and endosperm development of the *naa15* mutant that had abnormal embryo differentiation and incomplete endosperm cellularization compared to WT of *Arabidopsis*, and comparatively investigated the changes of gene expressions in WT seeds at 3, 4, and 5 days after pollination (3W, 4W, and 5W) and the white homozygous aborted *naa15* seeds at 5, 6, and 7 DAP (5M, 6M, and 7M) from *naa15-1*/+ siliques using RNA sequencing and qPCR assays. The transcriptome analyses showed that there were 2040 and 3630 differentially expressed genes (DEGs) in 4W (at endosperm cellularization initiation stage and heart embryo stage) vs 3W (at syncytium stage and globular embryo stage), and 5W (at end of endosperm cellularization stage and torpedo embryo stage) vs 4W, respectively. The KEGG and GO analyses showed that lipid metabolic processes and transmembrane transport related to cell wall biogenesis, cell division and differentiation, the plant hormone signaling pathway, photosynthesis, and transcription regulator activity were evidently enriched in WT and *naa15*. The heatmap and qPCR analyses showed that auxin response genes (*ARFs*), auxin transport genes (*PINs*) cytokinin synthesis genes (*LOGs*), cytokinin dehydrogenase genes (*CKXs*), cytokinin receptor, transcription factors (*MYB*, *bHLH*, *MADS-box*, and *ERF*) were significantly downregulated in *naa15* compared to WT. A series of cell wall genes annotated to xyloglucan endotransglycosylase/hydrolase, pectin methyl esterase, and pectin methyl esterase inhibitor were also identified in these DEGs. Moreover, using an immunofluorescent assay, the features of cell walls displayed that cellulose fluorescence signals in the embryo and endosperm of *naa15* were significantly decreased, and the signals of low- and high- methyl esterification of pectin were also obviously decreased in the endosperm of *naa15*. In summary, we identified a large number of DEGs and investigated the features of cell walls during endosperm cellularization and embryonic differentiation, which provided important information on transcription and gene expression to reveal their regulatory mechanisms.

## Introduction

Seed development in angiosperms is triggered by double fertilization, in which sperm cells reach the embryo sac, and then two sperm cells fuse with egg cell and central cell resulting in the formation of diploid embryo and triploid endosperm, respectively (Dresselhaus et al., 2016; Wang et al., 2022). The intact seed contains the embryo, endosperm, and seed coat (Lafon-Placette and Köhler, 2014). In *Arabidopsis*, the embryo undergoes a series of cell division and organ differentiation, leading to its patterning establishment and maturation. The embryo reaches the pre-globular stage after eight rounds of cell divisions. Meanwhile, the endosperm conducts rapid and successive free nuclear divisions without cell wall formation and enters the syncytial endosperm phase (Sørensen et al., 2002). With the embryo development transition from the globular to heart stage, endosperm cellularization begins from the micropylar zone near the developing embryo with cell wall formation and moves forward the chalazal pole of seed. When the embryo enters torpedo phase, endosperm completes cellularization process (Berger, 2003; Li et al., 2013). When embryo develops from heart to torpedo stage, chloroplast biogenesis initiates and embryo turns green (Mansfield and Briarty, 1991). Endosperm cellularization process is an important milestone in endosperm development and embryogenesis, and it proceeds gradually with embryo differentiation (Wang et al., 2022).

The regulatory mechanisms of embryo differentiation and endosperm cellularization are sophisticated and involve in multiplex pathways of gene regulation. The *apetala2* (*ap2*) mutant endosperm undergoes an early expanded growth period to lead to delayed endosperm cellularization and overgrowth of the endosperm central vacuole (Ohto et al., 2009). MINI3 (WRKY10), a WRKY-type transcription factor, is specifically expressed in endosperm and embryo, and its mutant has a precocious cellularization of endosperm and produces small seeds (Luo et al., 2005). AGAMOUS-LIKE proteins (AGLs) are a large family of MADS-box proteins that form heterodimers or homodimers among the MADS-box family. In the three mutants of *agl61*, *agl62*, and *agl80*, embryogenesis and endosperm cellularization are damaged to varying degrees (Portereiko et al., 2006; Kang et al., 2008; Steffen et al., 2008). In other transcription factors, the genes of the *ARF* and *bHLH* families are expressed in seeds, and their deletions also affect development processes of embryo and endosperm, leading to seed abortion (Sun et al., 2010).

Plant hormones, important signaling molecules, play vital roles during the entire plant lifecycle. Auxin is critical in embryo and endosperm development, and its biosynthesis is activated when sperm-egg fuse to form zygotes (Figueiredo et al., 2015). However, the change in auxin homeostasis affects its signal transmission in the seed coat and embryo, eventually leading to seed abortion. Excessive accumulation of auxin delays endosperm cellularization (Batista et al., 2019). In seed formation, auxin and cytokinin are related to cell division and differentiation, as well as organ growth and development (Bishopp et al., 2011; Vanstraelen and Benkova, 2012). The sizes of embryos and seeds are increased owing to disordered cytokinin homeostasis in *ahk2 ahk3 ahk4* mutants (Riefler et al., 2006). Brassinosteroids are essential steroid hormones that play critical roles in plant reproductive growth and seed development. In the rice BR-deficient mutant *brd2* and *Arabidopsis* BR deficient mutants *dwf5* and *det2*, there are smaller seeds than WT (Hong et al., 2005; Jiang and Lin, 2013).

In addition to hormones, endosperm and embryo development are also related to cell wall properties. Methyl-esterified pectin is demethylesterified by pectin methylesterases (PMEs), which change the rigidity and stiffening of the cell wall in accordance with the pattern of dimethyl esterification (Levesque-Tremblay et al., 2015).

In 5-6 DAP *Arabidopsis brd-1* mutant, the fluorescence signal of low-esterified homogalacturonic acids (HGs) in endosperm cell wall are faint and heterogeneous, but the signal of high-esterified HGs significantly increases (Cruz-Valderrama et al., 2018). In rice, there are seven predicted invertase/PME inhibitor genes, which are exclusively expressed during seed development (French et al., 2014). The overexpression of the pectin methylesterase gene *PME5 in Arabidopsis* reduces the pectin methylesterification of flower primordia leading to the formation of ectopic primordia, whereas the overexpression of *pectin methylesterase inhibitor 3* (*PMEI3*) significantly increases the pectin methylesterification, which inhibits organ formation (Peaucelle et al., 2011). In the *Arabidopsis zou-4* mutant, the seeds retain persistent endosperm growth to lead to abnormal increases in seed size, and the embryo sheath that is located near the surface of embryo at the late heart stage cannot be detected with a JIM12 antibody (Fourquin et al., 2016; Moussu et al., 2017).

N-terminal protein α-acetylation (NTA) is a highly prevalent protein modification that affects multiple cellular functions, such as cell multiplication, regulation of stress stimulation, and immune response (Linster et al., 2015; Xu et al., 2015; Giglione and Meinnel, 2021). Plastids are one of the places where co- and post-translational N-terminal acetylation occurs, and approximately 20-30% of all plastid proteins are affected by NTA (Bischof et al., 2011; Bienvenut et al., 2012). The process of N-terminal acetylation is that N-terminal acetyltransferases (NATs) transfer acetyl groups of acetyl-CoA to α amino acids of nascent polypeptides with 25-50 amino acids (Gautschi et al., 2003; Varland et al., 2015). N-terminal acetyltransferase A (NatA), one of the Nats (NatA to NatF), consists of the catalytic subunit NAA10 and the auxiliary subunit NAA15, and is the predominant acetyltransferase complex in yeast and humans (Arnesen et al., 2009). The auxiliary subunit NAA15 anchored to the ribosome participates in acetyltransferase activity, and/or interacts with nascent polypeptides (Ree et al., 2018).

In *Arabidopsis*, a point mutation of *NAA15* leads to immune receptor suppression of NPR1 protein instability and decreases plant immunity (Xu et al., 2015), and T-DNA insertion mutants of *NAA10* and *NAA15* in *Arabidopsis* display embryo abortion and abnormal endosperm (Feng et al., 2016; Chen et al., 2018a). In humans, truncating variants of NAA15 lead to varying degrees of growth defects, including intellectual disability and autism spectrum disorder (Cheng et al., 2018). The artificial microRNAs against *NAA10* and *NAA15* significantly downregulate their transcripts and show drought stress tolerance by activation of the abscisic acid response in *Arabidopsis* (Linster et al., 2015). However, the transcriptional regulation of *NatA* during embryo differentiation and endosperm cellularization in *Arabidopsis* is unknown to date. Here, *Arabidopsis* seeds from WT and *naa15* at three developmental stages were used for transcriptomic analyses and gene expression assays. Meanwhile, the cell wall features were analyzed by detecting the degrees of pectin methyl esterification and cellulose. In our transcriptome profile, a list of genes associated with plant hormones were also identified and analyzed. These results contributed to understanding the transcriptional regulatory information and network relationship during embryo differentiation and endosperm cellularization.

## Materials and methods

### Plant material and growth conditions

*Arabidopsis thaliana* ecotype Columbia-0 (Col-0) was used as wild-type plant (WT), and the T-DNA insertion mutant *naa15-1/+* (CS836292) was used as described previously (Chen et al., 2018a). All plants were cultivated at 22 ± 2°C with 16-h light/8-h dark in a growth chamber of Wuhan University. The genotype assay of *naa15/+* was shown in **Supplementary Figure 1** by using PCR amplification, and the gene specific primers were listed in **Supplementary Table 1**.

### Observation of cleared seeds and cellularization endosperm

Seeds were quickly and carefully dissected from siliques and then cleared in Hoyer’s solution (chloral hydrate: glycerol: water, 8:1:2) 12 hours at 4°C (Chen et al., 2018a). The transparent seeds were placed onto slides and observed under a Leica SP8 microscope (Leica, Germany) equipped with differential interference contrast (DIC) optics. To observe endosperm cellularization, the seeds were fixed in 4% glutaraldehyde in PBS (pH 7.0) under vacuum conditions for 1 h and then transferred into freshly 4% glutaraldehyde in PBS (pH 7.0) and incubated overnight at 4°C. The samples were progressively dehydrated according to a series of alcohol gradients, 15%, 30%, 50%, 70%, 90%, 100%, and then rehydrated using alcohol gradients of 90%, 70%, 50%, 30%, 15%, each gradient for 20 min. The samples were cleared with Hoyer’s solution overnight at 4°C, and observed under a Leica TCS SP8 confocal microscope. The excitation and emission wavelengths were 488 and 505–535 nm, respectively (Li et al., 2017).

### RNA isolation and Illumina sequencing

For RNA-seq material, WT seeds at 3, 4, and 5 days after pollination (DAP) (3W, 4W, and 5W) and the homozygous white aborted *naa15* seeds at 5, 6, and 7 DAP (5M, 6M, and 7M) were separated under dissecting microscope, respectively. Transcriptome sequencing was performed by Majorbio Biotech Co., Ltd. (Shanghai, China). For RNA-sequencing (RNA-seq), the total RNA of samples was extracted using an OminiPlant RNA Kit (CWBIO, Beijing, China) and digested with DNaseI. RNA quality was analyzed by 1.2% agarose gel electrophoresis. The mRNA was purified using poly-T-oligo-attached magnetic beads. The purified mRNA was broken into 300 bp (± 20 bp) paired-end reads. Raw data were obtained and analyzed on an Illumina HiSeq 2500 platform. More than 6.73 Gb of high-quality reads were generated from each cDNA library.

### Gene sequence assembly and functional annotations

To obtain high-quality clean reads, we removed some reads with over 10% uncertain bases (N) and the low sequencing quality reads of the raw reads were also cleared. Transcriptome assembly was completed using Cufflinks 2.2.1 (http://cole-trapnell-lab.github.io/cufflinks/) and StringTie software (http://ccb.jhu.edu/software/stringtie/). Principal component analysis (PCA) of RNA-seq samples was implemented to detect the expression variances using the plotPCA function in RSEM software (http://deweylab.github.io/RSEM/). Gene functions were annotated using the NR, Swiss-Prot, Pfam, EggNOG, GO, and KEGG databases.

### Identification of differentially expressed genes

The sequencing reads were aligned to the Arabidopsis TAIR 10.0 reference genome (http://plants.ensembl.org/Arabidopsisthaliana/Info/Index) using HISAT2 (http://ccb.jhu.edu/software/hisat2/index.shtml). The expression values of each transcript were calculated with the Fragments Per Kilobases per Million reads (FPKM) method. Differentially expressed genes (DEGs) were identified by comparing the gene expression levels between different groups using the DESeq R package. A log_2_ (Fold Change) ≥ 1 or ≤ −1 and P-value < 0.05 were set as the thresholds to discriminate DEGs. Gene Ontology (GO) and Kyoto Encyclopedia of Genes and Genomes (KEGG) enrichment analyses were performed to investigate the putative functions of DEGs. The top 30 terms of GO enrichment analysis of enriched DEGs were counted with the enriched P-value < 0.05. Venn diagrams were constructed using Venny 2.1 (http://bioinfogp.cnb.csic.es/tools/venny/) to obtain the intersection targets. The hierarchical clustering of heatmap analyses was generated using the online tool (http://www.heatmapper.ca/expression/) (Babicki et al., 2016). Pairwise distances were measured using Pearson correlation, and average linkage was set as the clustering method in heatmap analyses (Karunakaran et al., 2021; Lemke et al., 2021).

### Validation of DEGs by qPCR

Total RNA was extracted using an OminiPlant RNA Kit (CWBIO, Beijing, China), and cDNA was synthesized using an EasyScript^®^ One-Step gDNA Removal and cDNA Synthesis SuperMix Kit (TransGen, Beijing, China). Quantitative real-time PCR (qPCR) was conducted using Transtart Top Green qPCR SuperMix (TransGen Biotech, Beijing, China) and automatically analyzed by the Bio-Rad CFX Manager 3.1 system (Bio-Rad, USA). *AtGAPDH* was used as an internal reference to normalize the gene expression levels. The specific gene primers were designed by Primer Premier 5.0 and were listed in **Supplementary Table 1**. For qPCR analysis, the sample was independently repeated in triplicate, and the relative expression values were calculated by the delta-delta Ct (ΔΔCT) method as described previously (Li et al., 2017).

### Semithin sections, histological and immunohistochemical analyses

For semithin sections, seeds of WT and the white aborted seeds of *naa15/+* were fixed in 4% (v/v) paraformaldehyde buffered with 100 mM phosphate buffered saline (PBS, pH 7.4), placed under vacuum for 30 min, and then fixed overnight at 4°C. After fixation, the samples were washed four times with PBS. The samples were gradually dehydrated in a series of alcohol gradients (Zou et al., 2021). After dehydration, the samples were embedded in LR-White resin and polymerized at 50°C for two days. The samples were cut into sections with 1 μm thickness under an ultramicrotome (EM UC7, Leica) and then placed on slides.

To observe cell wall features of seeds, the samples were incubated in a mixture of calcofluor white (Sigma−Aldrich, St. Louis, USA) and 10% KOH (1:1, v/v) for 5 min at room temperature, and their fluorescent signals were observed under a Leica SP8 microscope. For immunofluorescent analysis of seeds, the samples were washed with distilled water and treated with the blocking solution (5% BSA buffered with PBS, pH 7.4) for 3 h. The primary rat monoclonal antibodies John Innes Monoclonal 5 (JIM5, antibody of low methyl-esterified HGs) and JIM7 (antibody of high methyl-esterified HGs) (diluted 1:10 in blocking solution) were added to the semithin sections at 4°C for 12 h. After incubation, the sections were washed with PBS solution three times to remove the primary rat antibody solution (Ma et al., 2019). The fluorochrome secondary antibody anti-mouse IgG DyLight 488 (Abbkine, Beijing, China) was added, and the sections were covered for 2 h at room temperature. Finally, the samples were observed under a Leica SP8 microscope with the same microscope magnification and fluorescence intensity. Immunofluorescence assays were conducted under the same conditions. The fluorescence signals were quantitated under a Leica LAS AF Lite v2.6.3. The fluorescence intensity of the cell walls was measured at five to ten points in each seed, and the final value was an average represented by a black dot.

### Statistical analysis

All statistical tests and graphic plotting were performed using GraphPad Prism Software version 8.0 (GraphPad, San Diego, USA). Values were expressed as the mean ± standard deviation (SD). The experiments were conducted with three independent biological replicates. Statistical significance was established according to Student’s *t*-test analysis, and the significance levels were established at *P<0.05, **P<0.01, and ***P < 0.001.

## Results

### Phenotypic characteristics of embryo differentiation and endosperm cellularization in WT and *naa15*

**Figure 1:**
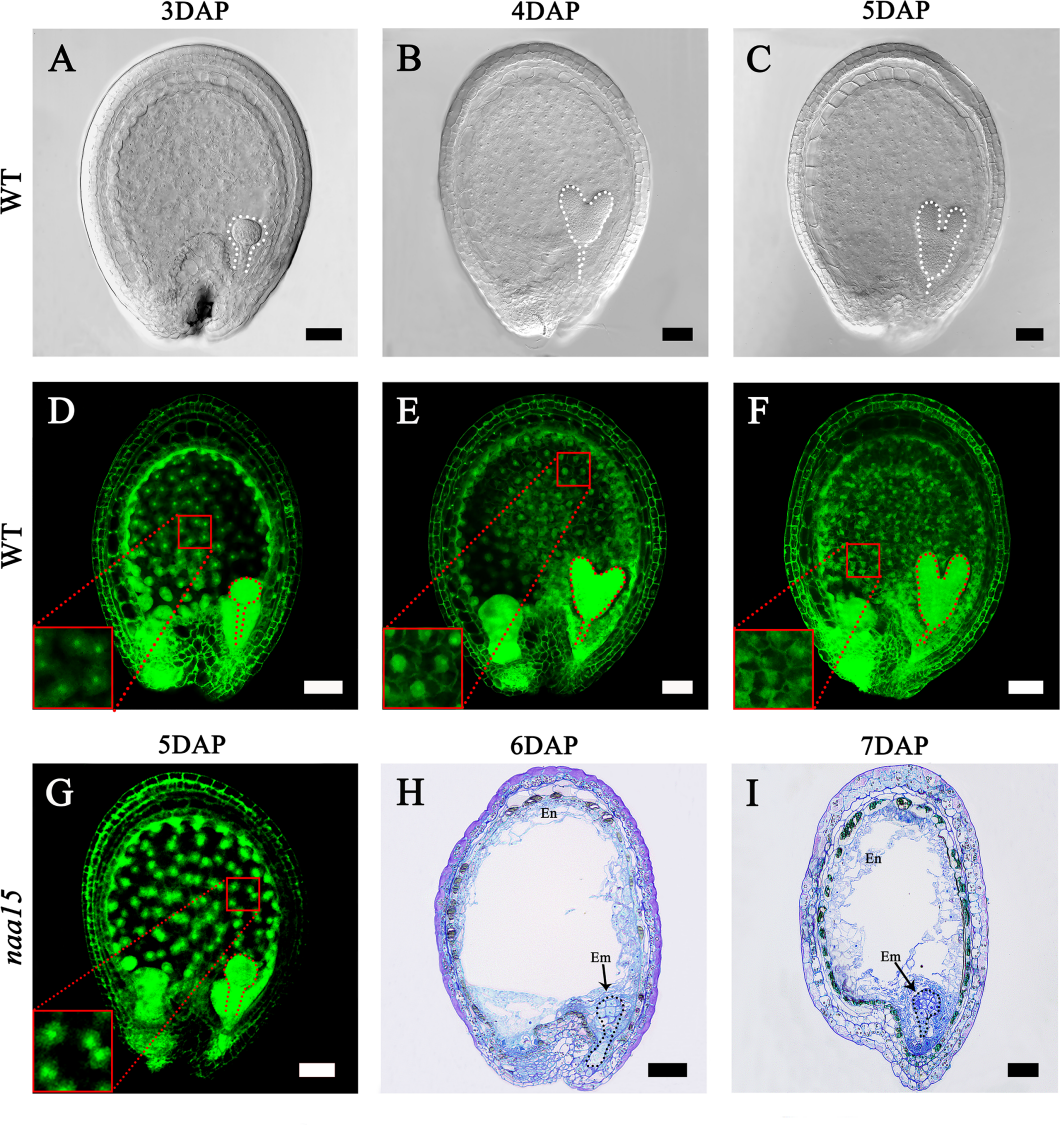
Embryo differentiation and endosperm cellularization in WT and *naa15* of *Arabidopsis*. **(A–F)** Seeds in WT; **(G-I)** Seeds in *naa15*. **(A)** A seed with a globular embryo at 3 DAP. **(B)** The seed with a heart embryo at 4 DAP. **(C)** The seed with a torpedo embryo at 5 DAP. **(D)** Free nucleus dispersed in syncytium endosperm at 3 DAP. **(E)** Free endosperm nucleus began to form cell wall at 4 DAP. **(F)** Endosperm cellularization at 5 DAP basically completed. **(G)** The seed showed an abnormal globular embryo with free endosperm nucleus without cell walls at 5 DAP. **(H, I)** Semi-thin sections showed abnormal globular embryo and partially endosperm cells at 6 DAP and 7 DAP, respectively. Embryos were marked with discontinuous lines of different colors. The small red boxes were magnified and shown on the lower left of the corresponding images. Em, embryo; En, endosperm. Scale bars = 50 μm.

The embryo and endosperm are the most important components of seeds. In our early research, we obtained an *Arabidopsis naa15* mutant that possesses the deficient embryo and endosperm (Chen et al., 2018a). To investigate the characteristics of endosperm cellularization and embryo differentiation in *Arabidopsis* seeds, we observed the development processes of seeds at different stages. In 3W, the embryo reached the globular stage, and the endosperm presented dispersed free nuclei (**Figures 1A, D**). Subsequently, the embryos developed to the heart and torpedo stages at 4 DAP and 5 DAP, respectively (**Figures 1B, C**). In 4W, the cell wall of free endosperm nuclei formed in the middle and micropylar end of seeds, indicating that the endosperm was undergoing a process of cellularization (**Figure 1E**). In 5W, the cell walls of the free endosperm nuclei around the chalazal end were formed, which implied that the endosperm basically completed the process of cellularization (**Figure 1F**). We observed the embryo and endosperm phenotypic features of the *naa15* mutant using spontaneous fluorescence detection and semithin sections. In 5-7M, embryos stagnated at abnormal globular stages (**Figures 1G–I**), indicating that embryo differentiation was severely disrupted. In 5M, the endosperm was still in the syncytial stage, showing that its cellularization failed to be normally initiated. In 6 and 7 M, the partial free endosperm nuclei formed cell walls, but seeds produced cavities due to abnormal cellularization. The results indicated that the mutation of *NAA15* led to incomplete endosperm cellularization (**Figures 1H, I**). Therefore, we called this abnormal development occurring in the embryo and endosperm of the *naa15* seeds as “Globular embryo abnormal development” and “Endosperm delayed development”. Based on the phenotypic characteristics of endosperm and embryo development described above, the 5M had an abnormal globular embryo and syncytial endosperm (**Figure 1G**), which was similar to the 3W. The 6M formed the partial endosperm cell wall and was similar to 4W (**Figure 1H**). The 5W completed cellularization, but the *naa15* mutant still had a large cavity and incomplete cellularization. Therefore, the *naa15* mutant was a well-experimental material for investigating the transcriptional regulation and gene expression in embryo differentiation and endosperm cellularization of *Arabidopsis*.

### Global analysis of the RNA-seq data

The seeds of 5 DAP WT turned green from translucent, while the *naa15* seeds at 5DAP were still translucent and white in *naa15*/+ siliques (**Supplementary Figure 1A**). To investigate the transcriptional levels during endosperm cellularization and embryo differentiation, we collected normal seeds of 3-5 DAP WT (3W, 4W, and 5W, respectively) and white aborted homozygous seeds of 5-7 DAP *naa15* (5M, 6M, and 7M, respectively) as the tested materials and conducted RNA sequencing. The results showed that a total of 137.14 Gb of clean reads was obtained, and the mapping rate to the *Arabidopsis* genome was over 97%. The statistics of the clean reads in each sample were listed in **Supplementary Table 2**. The FPKM values of genes were shown in **Supplementary Table 3** and the gene expression levels in each library were calculated and represented as the log_10_ (FPKM) method (**Supplementary Figure 2A**). The principal components of the PCA score plot (PC1 and PC2) were calculated to be 43.76% and 16.22%, respectively (**Supplementary Figures 2B, C**). The six sample groups were clearly separated, and three biological replicates of each group were clustered together (**Supplementary Figure 2C**).

**Figure 2:**
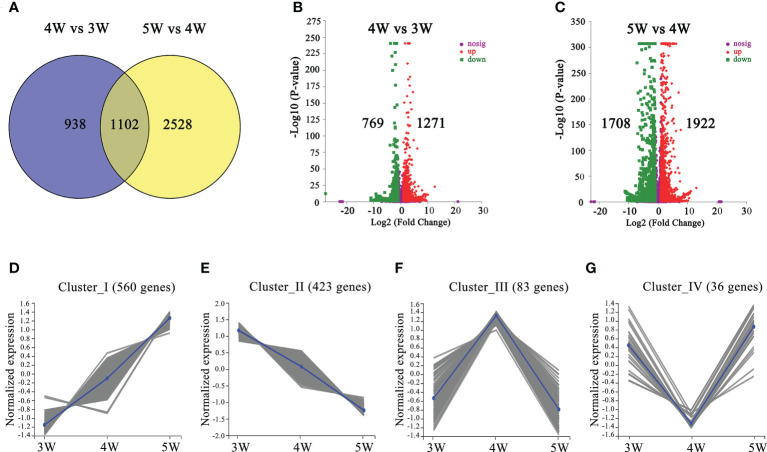
The number and functional annotation of differentially expressed genes (DEGs) during embryo differentiation and endosperm cellularization in WT of *Arabidopsis*. **(A)** The number of DEGs from venn diagram analysis in 4W vs 3W and 5W vs 4W groups, respectively. **(B, C)** Volcano maps of DEGs in 4W vs 3W and 5W vs 4W groups, respectively. Log_2_ (Fold Change)≥ 1 or ≤ −1, and P-value < 0.05 were used as a standard for screening DEGs. The “-Log_10_ (P-value)” indicated the statistical significance level, and the larger value showed that the divergence of gene expression was more significant. Significantly up- or down-regulated genes were represented by using red dots or green dots, respectively. **(D–G)** Clustered gene expression profiles of 1102 DEGs from 4W vs 3W and 5W vs 4W using the statistical method of K-means.

We performed comparisons between 4W vs 3W (heart stage vs globular stage) and 5W vs 4W (torpedo stage vs heart stage) to uncover the changes in the transcriptome profiles during the early stages of embryo and endosperm development. A total of 2040 DEGs (1271 up- and 769 down-regulated) in 4W vs 3W and 3630 DEGs (1922 up- and 1708 down-regulated) in 5W vs 4W groups were obtained, respectively (**Figures 2B, C**). Venn diagrams showed that 1102 DEGs were shared between the 4W vs 3W and the 5W vs 4W groups (**Figure 2A**), and these DEGs were distributed in four clusters using the K-means approach, indicating that they had similar expression patterns in 3-5 DAP WT (**Figures 2D-G** and **Supplementary Tables 4**-**7**). Many transcription factors, such as growth-regulating proteins, MYBs, and bZIPs were in cluster I (560 DEGs), showing that their expression levels were distinctly upregulated with seed development (**Figure 2D** and **Supplementary Table 8**). In contrast, the transcript levels of auxin response factors (*ARF12*, *ARF13*, *ARF14*, *ARF15*, *ARF20*, *ARF21*, and *ARF22*) and MADS-box transcription factors (*AGL36*, *AGL48*, *AGL58*, *AGL62*, *AGL90*, and *AGL 96*) were significantly downregulated from 3W to 5W, and were all clustered in cluster II (423 DEGs) (**Figure 2E** and **Supplementary Table 9**). The numbers of DEGs in clusters III and IV were 83 and 36, respectively. The genes involved in heat shock proteins and chaperones were enriched in cluster III, while many pectin acetylesterase, methyltransferase, and protein kinase enzymes were concentrated in cluster IV (**Supplementary Tables 6**, **7**). Therefore, we proposed that the differential transcriptional regulation of genes may contribute to early seed development.

### Functional enrichment analysis of DEGs based on KEGG and GO databases

**Figure 3:**
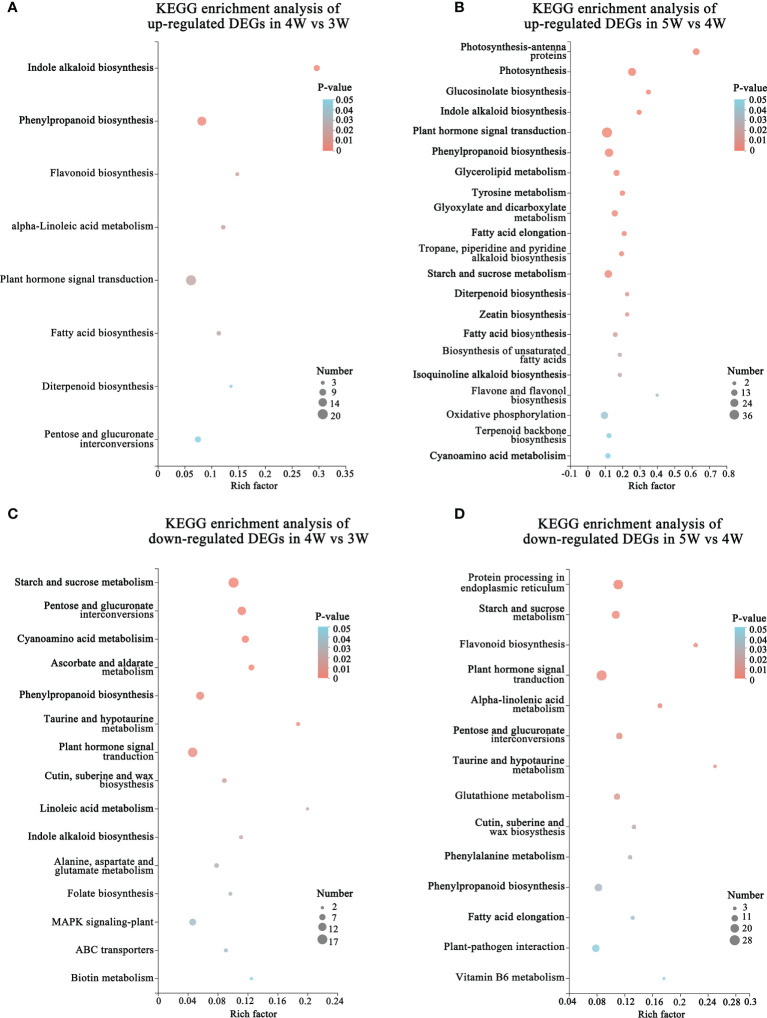
Functional annotation and classification of DEGs during embryo differentiation and endosperm cellularization in WT of *Arabidopsis*. **(A, B)** KEGG enrichment analysis of up-regulated DEGs in 4W vs 3W and 5W vs 4W groups, respectively. The “Rich factor” represented the enrichment degree of DEGs in each pathway. The number of the enriched DEGs was represented by the size of each circle. The larger the number, the bigger the circle. **(C, D)** Diagrams of KEGG enrichment analysis of down-regulated DEGs in 4W vs 3W and 5W vs 4W, respectively.

To reveal the regulatory network of seed morphogenesis, DEGs were classified and annotated using GO and KEGG databases. The up-regulated DEGs of KEGG enrichment contained indole alkaloid biosynthesis, plant hormone signal transduction and phenylpropanoid biosynthesis in the 4W vs 3W and 5W vs 4W groups (**Figures 3A, B** and **Supplementary Table 10**). A lot of terms related-cell division, the RNA metabolism, the transcription regulator activity, cell cycle terms, and cytoskeleton-related terms, were significantly enriched in up-regulated DEGs of 4W vs 3W (**Supplementary Figure 3A**), indicating that 3 to 4 DAP seeds might undergo vigorous cell divisions. The photosynthesis, photosynthesis - antenna proteins, starch and sucrose metabolism, and oxidative phosphorylation pathways were significantly enriched in 5W vs 4W (**Figure 3B**). Within the biological process category of GO enrichment analyses, many up-regulated DEGs were also significantly enriched in photosynthesis terms (GO:0019684, GO:0009768, and GO:0015979) (**Supplementary Figure 3B**), indicating that they might participate in plastid transformation to the chloroplast process during early seed development and involve in the embryonic transfer from the heterotrophic stage to the autotrophic stage.

In addition, the terms related to cell wall (cell wall organization or biogenesis, plant-type cell wall organization or biogenesis, and cell wall) were also significantly enriched in cellular component using GO enrichment analysis of 4W vs 3W and 5W vs 4W groups (**Supplementary Figures 3B,C**), which mainly included the genes related to cell wall modification (pectinesterase and pectin methylesterase inhibitor), cellulose synthase, and expansins (**Supplementary Table 11**). The hormone signaling pathways were significantly enriched in up- and down-regulated DEG of GO enriched terms and KEGG enriched pathways during early seed development at 3-5 DAP (**Figures 3A–D**). These DEGs mainly participated in the auxin, cytokinin, and abscisic acid pathways, indicating that there was wide crosstalk among hormones. The mRNA levels of auxin transport genes (*PIN6* and *LAX2*), auxin response factors (*ARF4* and *ARF11*), and small auxin upregulated RNA 78 (*SAUR78*) were markedly upregulated from 3 to 5 DAP. Particularly, cytokinin synthesis genes *LONELY GUY* 4 and 5 (*LOG4* and *LOG5*) were mainly upregulated in 4W vs 3W (**Supplementary Table 11**), speculating that they might be involved in the formation of embryo organ primordia and cellularization of free endosperm nuclei.

### Candidate regulation pathways during endosperm cellularization and embryo differentiation of *naa15*

**Figure 4:**
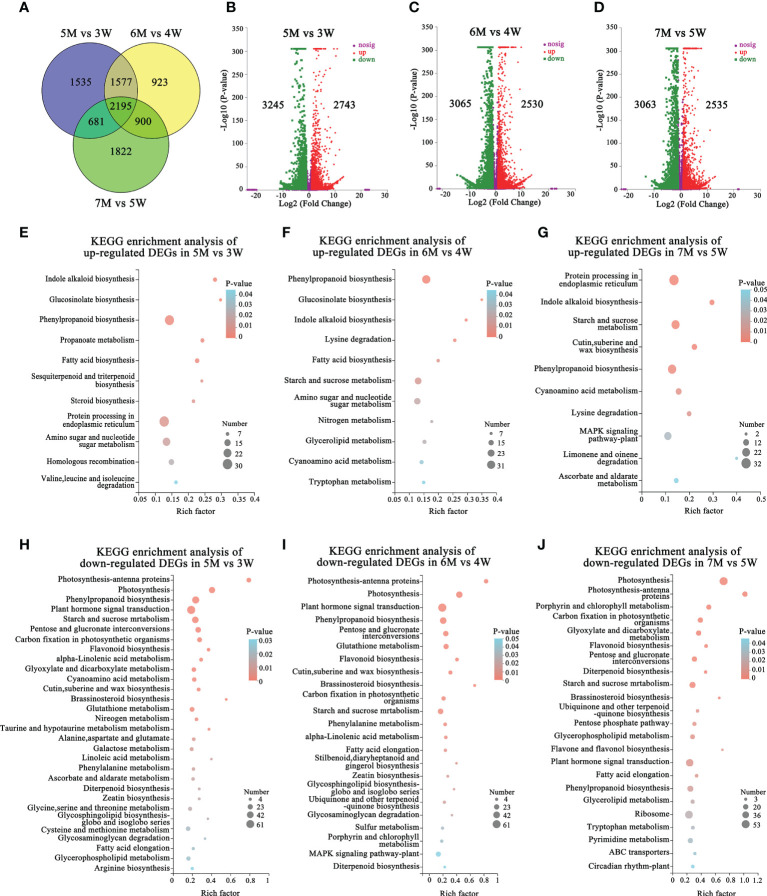
The number and KEGG enrichment pathways of DEGs between *Arabidopsis* WT and *naa15* in 5M vs 3W, 6M vs 4W, and 7M vs 5W, respectively. **(A)** Venn diagram of DEGs in three comparison groups between WT and *naa15*. **(B–D)** The volcano maps of DEGs in three comparison groups between WT and *naa15*. **(E–G)** The KEGG enrichment pathways of up-regulated DEGs in 5M vs 3W, 6M vs 4W, and 7M vs 5W, respectively. **(H–J)** The KEGG enrichment pathways of down-regulated DEGs in 5M vs 3W, 6M vs 4W, and 7M vs 5W, respectively.

We performed the transcriptome comparative analysis in both WT and *naa15*, and identified a total of 9633 DEGs in 5M vs 3W, 6M vs 4W and 7M vs 5W. Among these DEGs, the 2195 DEGs were shared in three comparison groups (**Figure 4A**). The number of DEGs was 5988 DEGs in 5M vs 3W (containing 2743 up- and 3245 down-regulated), 5595 DEGs in 6M vs 4W (containing 2530 up- and 3065 down-regulated), and 5598 DEGs in 7M vs 5W (containing 3063 up- and 2535 down-regulated), respectively (**Figures 4B–D**). KEGG enriched analysis showed that the up-regulated DEGs of WT and *naa15* were significantly enriched in phenylpropanoid biosynthesis, protein processing in endoplasmic reticulum, starch and sucrose metabolism, and amino sugar and nucleotide sugar metabolism (**Figures 4E–G** and **Supplementary Tables 12A–C**). The GO enrichment analysis displayed that the up-regulated DEGs of WT and *naa15* were evidently aggregated in the terms of cell cycle process, cell division, cytoskeleton part, and related-microtubule terms (including microtubule binding, microtubule-based process, and microtubule-based movement) (**Supplementary Figure 6**).

**Figure 5:**
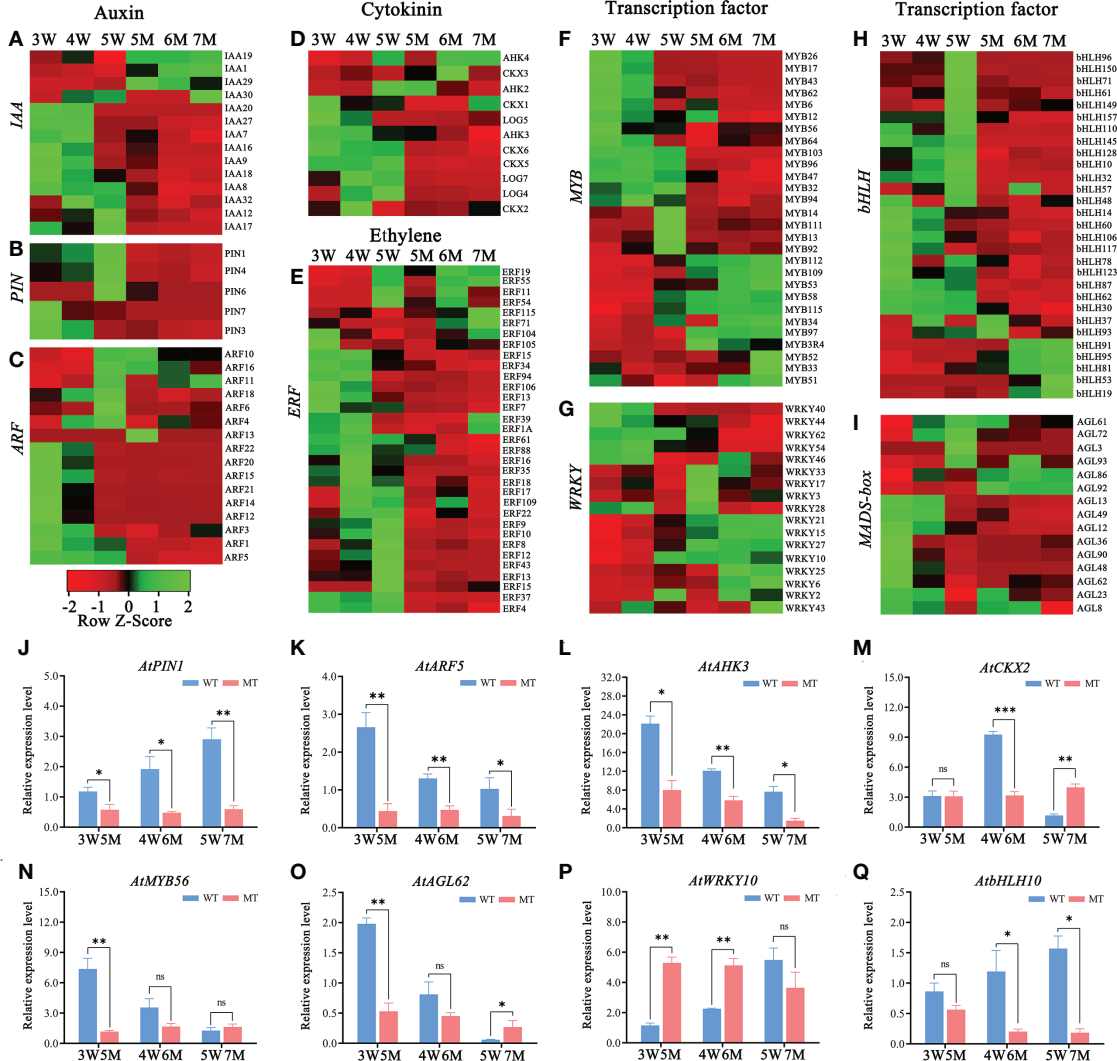
Heatmaps and qPCR analyses of DEGs related to hormones and transcription factors in WT and *naa15* seeds of *Arabidopsis*. **(A–D)** The relative expression patterns of DEGs involved in auxin and cytokinin signal pathways. **(E–I)** The relative expression patterns of DEGs involved in transcription factors including ERF, MYB, WRKY, bHLH, and MADS-box. FPKM values were standardized with Z-Score for the heatmap analysis. Red represented down-regulation of gene expression, and green represented up-regulation of gene expression. **(J–Q)** The qPCR analyses of DEGs related to hormones and transcription factors. Asterisks denoted significant differences basing on Student’s *t*-test (*P<0.05, **P<0.01, and ***P < 0.001).

The down-regulated DEGs of KEGG enrichment analyses in WT and *naa15* were mainly enriched in plant hormone signal transduction photosynthesis, phenylpropanoid biosynthesis, and starch and sucrose metabolism pathways (**Figures 4H–J** and **Supplementary Tables 12D–F**). Totally, we identified 137 DEGs involved in auxin, cytokinin, and ethylene signaling pathways in three comparison groups between WT and *naa15* (**Supplementary Table 13**). As shown in **Figures 5A–C**, the genes annotated to auxin-responsive proteins (IAA), PIN, and auxin response factors (ARF) were identified, and most of them were downregulated in *naa15*. qPCR analyses showed that the expression of auxin transporter *PIN1* was significantly increased in 3W-5W. In contrast, the *PIN1* transcript level was markedly decreased in *naa15* (**Figure 5J**). The mRNA levels of *ARF5* showed a downregulated pattern in WT and were decreased in 5-7M (**Figures 5C, K** **)**.

In addition, the heatmap showed that the genes related to the cytokinin pathway, cytokinin receptors (*ARABIDOPSIS HISTIDINE KINASE*, *AHK3*), cytokinin dehydrogenases (*CYTOKININ OXIDASE/DEHYDROGENASE*, *CKX1*, *CKX2*, *CKX5*, and *CKX6*), and cytokinin-activating enzymes (*LONELY GUY*, *LOG4*, *LOG5*, and *LOG7*) were also downregulated in *naa15* (**Figure 5D**). The cytokinin receptor *AHK3* in 3-5 W was gradually downregulated, while its transcript was distinctly decreased in 5-7 M (**Figure 5L**). *CKX2*, encoding a limiting enzyme in cytokinin metabolism, is activated by WRKY10 to promote endosperm growth (Li et al., 2013). In our results, the expression of *CKX2* was upregulated at 3W and 4W, but down-regulated at 5W; however, it remained at low levels in 5-7M (**Figure 5M**). These results implied that the gene expression of *NAA15* was involved in regulating the hormone signaling pathways during embryo and endosperm development.

Transcription factors (TFs), including MYB, bHLH, WRKY, ERF, and GATA, were identified in GO and KEGG enriched analyses in comparison between WT and *naa15*. In 5M vs 3W group, there were 89 transcription factors (TFs) (53 down- and 36 up-regulated DEGs), and the most downregulated TFs was the bHLH family (18 DEGs), followed by ERF family (16 DEGs). In 7M vs 5W group, the downregulated TFs were mainly in the MYB, ERF, and bHLH families (**Supplementary Table 14**). The heatmaps showed that ERF (23 DEGs), MYB (16 DEGs) and bHLH (35 DEGs) were significantly down-regulated in 5-7M (**Figures 5E, F, H**). The gene expressions of *WRKY* (17 DEGs) and *AGL* (15 DEGs) were also significantly changed in 5-7M (**Figures 5G, I**). qPCR analyses showed that the expressions of *AtMYB56* and *AtAGL62* gradually decreased in 3-5W, and their expressions distinctly deceased in 5M compared to WT (**Figures 5N, O**). *AtbHLH10* and *AtWRKY10* were markedly increased in 3-5W (**Figures 5P, Q**). Nevertheless, *AtWRKY10* in 5 and 6M had higher expression than in 3W and 4W (**Figure 5P**). Hence, we hypothesized that *NAA15* impacted embryo differentiation and endosperm cellularization by directly or indirectly mediating the transcription levels of these genes.

Sucrose controls a wide range of developmental and metabolic processes including cell division, cotyledon development, far-red light signaling, and tuber development (Yoon et al., 2021). In our research, the starch and sucrose metabolism pathway was enriched in the KEGG enriched analysis of 3W-5W (**Figures 3B–D**). And the expressions of two sucrose synthetases (*suc1* and *suc2*) and eight sucrose transporter genes (*sweet3*, *sweet4*, *sweet5*, *sweet7*, *sweet9*, *sus1*, *sus6*, and *sus4*) were significantly decreased in *naa15* (**Supplementary Figure 4A**). Sucrose stimulates the expression of transcription factors such as *WRKY* and *MYB*, which function upstream of sucrose-responsive genes. However, the molecular mechanism of sucrose as a signal regulating embryo and endosperm development remains unknown. Our transcriptome data provided some related to sucrose genes that might play vital functions during early seed development.

The photosynthesis pathway was also significantly enriched in the KEGG and GO enriched analyses up-regulated DEGs in 5W vs 4W group (**Figure 3B** and **Supplementary Figure 3B**). The GO enrichment analysis of down-regulated DEGs in *naa15* was mainly enriched in photosystem and the relevant chloroplast genes (**Supplementary Figure 5**). The gene expressions from six *photosystem I-antenna* (*LHCI*) genes and 12 *chlorophyll a/b binding protein complex Ⅱ* were decreased in *naa15* (**Supplementary Figure 4B**). Interestingly, Lhca5, Lhcb1.1, PORB, and PORC have been considered potential substrates of *Arabidopsis* N-terminal acetyltransferase A (NatA), which consists of NAA10 and NAA15 (Linster et al., 2015). Hence, we speculated that plant hormones, photosynthesis, and carbohydrate pathways were critical during early seed development. However, the molecular regulatory function of *NAA15* in these pathways was unknown and requires further investigation.

**Figure 6:**
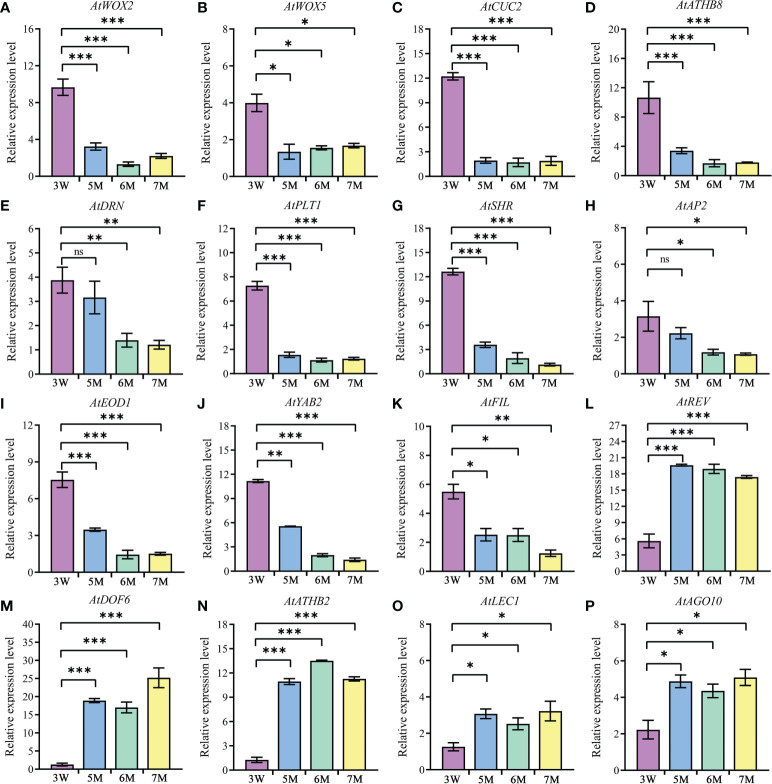
The expression of genes related to embryo differentiation in WT and *naa15*. **(A–P)** The qPCR analyses of DEGs in 3W, 5M, 6M, and 7M. 3W, 3 DAP WT; 5M, 5 DAP *naa15*; 6M, 6 DAP *naa15*; 7M, 7 DAP *naa15*. The "ns" represented no significant differences. Asterisks denoted significant differences basing on Student’s *t*-test *P < 0.05, **P < 0.01, and ***P < 0.001.

In addition, the abnormal globular embryo in *naa15* implied that embryo differentiation was disrupted. Thus, we inspected the expression levels of the genes reported to be associated with embryo differentiation in 3 DAP WT and 5-7 DAP *naa15*. WOX family had an essential role in body axis of embryo. Among these DEGs from our transcriptome profiles, *WOX2*, *WOX5*, and *WOX8* in *naa15* were significantly downregulated (**Supplementary Tables 2**, **4**), and the qPCR results also showed that the expressions of *AtWOX2* and *AtWOX5* in 5-7M were significantly decreased compared with that in 3W (**Figures 6A, B**), implying that the downregulated expression of WOX genes in *naa15* might lead to the formation of abnormal globular embryos. *CUP-SHAPED COTYLEDON* (*CUC*) and HD-ZIP TFs are involved in bilateral symmetry and SAM establishment. The qPCR results showed that *AtCUC2* and *ATHB8* were significantly decreased in *naa15* (**Figures 6C, D**). *DORNRÖSCHEN* (*DRN*) is an AP2/ERF-type TF that interacts with HD-ZIP III TFs. The expression of *AtDRN* also decreased significantly in *naa15* (**Figure 6E**). *PLETHORA1* (*PLT1*) and *SHORT-ROOT* (*SHR*) are required for hypophysis divided asymmetrically and quiescent center (QC) formation in late globular embryos, respectively (Aida et al., 2004).

In the results, *PLT1* and *SHR* showed high expressions at 3 DAP in WT, but down-regulated in 5-7M (**Figures 6F, G**), suggesting that it may be one cause of the abnormal globular embryo formation of *naa15*. Besides, the expressions of *APETALA2* (*AP2*) and *EOD1*, which influence the sizes of seeds, also significantly decreased in *naa15* (**Figures 6H, I**)*. YABBY2* (*YAB2*), *FILAMENTOUS FLOWER* (*FIL*), and *REVOLUTA* (*REV*) played essential roles in the establishment of abaxial patterning. In our study, the expressions of *YAB2* and *FIL* were significantly downregulated, while the *REV* was evidently upregulated (**Figures 6J-L**). In addition, *DOF6*, *ATHB2*, *LEC1*, and *AGO10* were observably up-regulated in *naa15* (**Figures 6M-P**), suggesting that the abnormal expression may affect the embryo differentiation of *naa15*. Therefore, we postulated that the gene expression changes related to embryo differentiation may be one of the causes of abnormal embryonic differentiation of *naa15*, and the detailed relationship between these DEGs and *NAA15* needs to be further investigated.

### Transcriptional regulation of cell wall formation and modification during early embryo and endosperm

**Figure 7:**
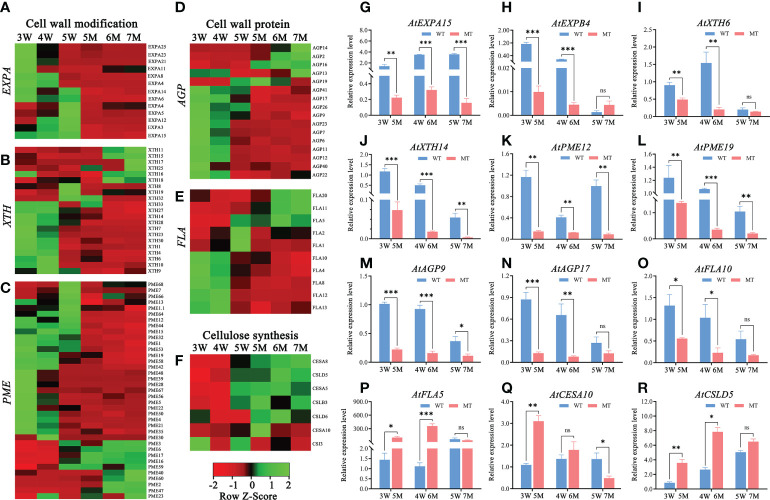
Heatmaps and qPCR analyses of DEGs related to cell wall formation between WT and *naa15* in *Arabidopsis* seeds at different development stages. **(A–C)** Heatmaps of the cell wall-modification proteins genes including the types of *EXP*, *XTH*, and *PME*/*PMEI*. **(D, E)** Heatmaps of the cell wall-protein genes including the types of *AGP* and *FLA*. **(F)** The heatmap of genes related to cellulose synthesis. **(G–R)** The qPCR analyses of DEGs related to formation of cell wall. Asterisks denoted significant differences basing on Student's t-test *P < 0.05, **P < 0.01, and ***P < 0.001. The "ns" represented no significant difference. AGP, arabinogalactan proteins; EXP, expansin; FLA, fasciclin-like arabinogalactan protein; PME/PMEI, pectinesterase/pectin methylesterase inhibitor; XTH, xyloglucan- endotransglycosylases/hydrolases.

According to GO and KEGG enrichment analyses from two comparison groups in 3-5W, many DEGs were enriched in cell wall-related pathways (e.g., cell wall organization or biogenesis and plant-typed cell wall) (**Figure 3** and **Supplementary Figure 3**). However, these DEGs were abnormal expressions in *naa15*. Heatmap analysis showed that 13 genes encoding expansin (EXPA), which functions as the modified proteins of cell wall, were downregulated in *naa15* (**Figure 7A**). The mRNA level of *AtEXPA15* was increased in 3W-5W, whereas it was significantly down-regulated in 5M-7M (**Figure 7G**). qPCR analyses showed that *AtEXPB4* was gradually downregulated in 3W-5W, and was also significantly downregulated in 5M and 6M compared to 3W and 4W (**Figure 7H**), hinting that the decrease in *AtEXPA15* and *AtEXPB6* mRNA levels in *naa15* might be one reason for abnormal endosperm cellularization. In addition, we also found that cell wall modification genes, including 15 xyloglucan endotransglycosylase/hydrolase (XTH) and 27 pectin esterase/pectin methylesterase (PME) genes, were downregulated in *naa15* (**Figures 7B, C**). qPCR analyses demonstrated that the expression of *PME12* and *PME19* in 5M were downregulated with 7.9-fold and 9.0-fold compared to 3W, respectively (**Figures 7K, L**). The expressions of *XTH6* and *XTH14* in 5M and 6M were downregulated compared to 3W and 4W (**Figures 7I, J**). The genes encoding proteins of cell wall, such as arabinogalactan proteins (AGPs, *AGP23/7/6/11/12/40/41/26/22/17/9*) and fasciclin-like arabinogalactan proteins (FLAs, *FLA10/4/8/12/13/2/1*), were also downregulated in *naa15* (**Figures 7D, E**). qPCR analyses confirmed that *AGP9*, *AGP17*, and *FLA10* were significantly downregulated in 5M and 6M compared to 3W and 4W (**Figures 7M–O**), which was consistent with the RNA sequencing data (**Figures 7D, E**). However, the expressions of several cell wall related-genes CESA5, CESA8, CSLD5, CSLB3, CSLD6, and FLA10 were up-regulated in naa15 (**Figures 7F**, **P**–**R**), which might play important role in abnormal cell walls of the endosperm.

### The features of cell walls during early embryo differentiation and endosperm cellularization

**Figure 8:**
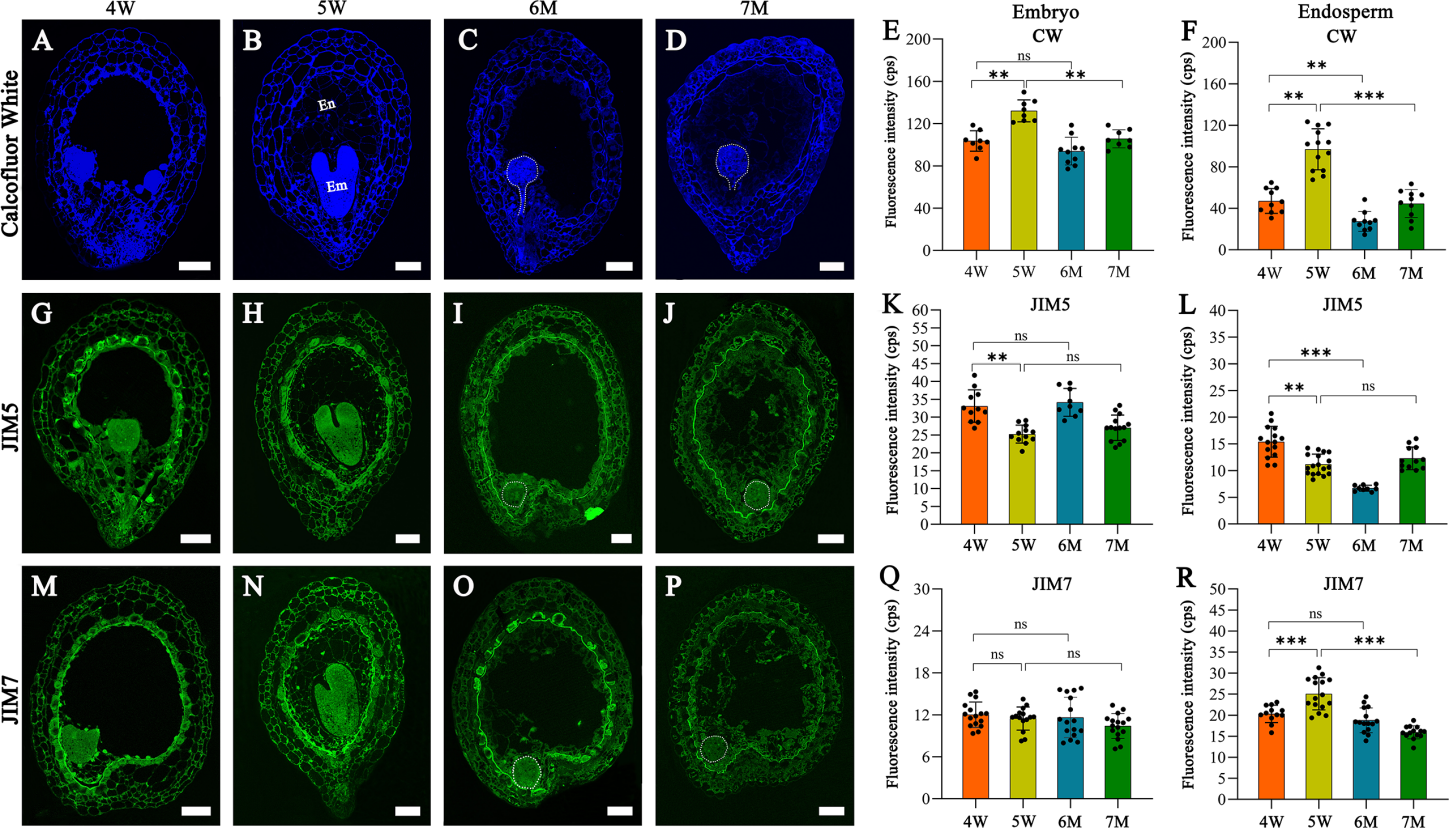
The images of histochemistry and immunofluorescence localization in WT and *naa15* seeds at different development stages. **(A–D)** Semi-thin sections of seeds were stained with calcofluor white in WT and *naa15*. **(E, F)** Fluorescence intensity with calcofluor white staining in embryo and endosperm of WT and *naa15*. **(G–J)** Semithin sections of seeds were immunolabeled using JIM5 antibody in WT and *naa15*. **(K, L)** Fluorescence intensity with JIM5 immunolabelling in embryo and endosperm of WT and *naa15*. **(M–P)** Semi-thin sections of seeds were immunolabeled using JIM7 antibody in WT and *naa15*. **(Q, R)** Fluorescence intensity with JIM7 immunolabelling of WT and *naa15* in embryo and endosperm, respectively. The p-value was calculated by using the Student *t*-test. Asterisks denoted significant differences, **P < 0.01 and ***P < 0.001. The "ns" represented no significant difference. Each black dot represented a seed. Em, embryo; En, endosperm; cps: counts per second. Scale bars = 50 µm.

Many DEGs from our transcriptome analysis were involved in cell wall formation and modification; therefore, we analyzed the features of cell wall in the embryo and endosperm of WT and *naa15*. Histological staining and immunofluorescence assays were performed in the seeds of 4W, 5W, 6M, and 7M using calcofluor white (CW) staining and pectin methyl-esterification monoclonal antibodies of (JIM5 and JIM7). The CW fluorescence gradually increased in cell walls of 4W and 5W embryos, while the signals in 7M embryonic cell walls significantly decreased compared to 5W (**Figures 8A–E**). The cell wall of *naa15* endosperm at 6M and 7M exhibited decreased fluorescence intensity compared to 5W (**Figures 8A–D, F**), inferring that the decreased cellulose in *naa15* might affect the formation of endosperm cell walls.

Using the JIM5 antibody to detect the low methyl-esterified pectin, the fluorescence signals of embryo cells decreased gradually from 4W to 5W and from 6M to 7M (**Figures 8G–K**). In cell walls of endosperm at 4W and 5W, JIM5 signals decreased following endosperm cellularization, which might contribute to the rapid development of subsequent embryos (**Figures 8G, H, L**). JIM5 fluorescence in the cell walls of *naa15* endosperm at 6M significantly decreased compared to 4W and 5W (**Figures 8G–J, L**), while the signal in 7M was similar to 5W. The results indicated the delayed and impaired endosperm growth and development in *naa15*

Using the JIM7 antibody to detect high methyl-esterified pectin, the results showed that there was no apparent change in the fluorescence signals in 6M and 7M compared to embryos at 4W and 5W (**Figures 8M–Q**). The fluorescence signals in the cell walls of endosperm at 4W and 5W were obviously increased, while the signal in 6M and 7M evidently decreased (**Figures 8M–P, R**), indicating that cell wall formation in embryo and endosperm of *naa15* was disrupted, resulting in the aberrant embryo differentiation and incomplete endosperm cellularization.

## Discussion

It is well known that seed formation depends on well-coordinated development between endosperm and embryo. The endosperm is considered as a nutrition source to support embryo development, and its cellularization process undergoes nuclear proliferation, formation of endosperm cell wall, and cell differentiation, all of which are essential for embryo growth (Jo et al., 2019; Song et al., 2021). Nevertheless, extensive efforts are required to understand the process of embryo differentiation and endosperm cellularization, including the fundamental regulatory mechanisms of the initial endosperm cellularization process, and the relationship between embryo and endosperm. In this research, we employed RNA-sequencing analyses and observed cell histological features to identify candidate genes and expression pathways involved in embryo differentiation and endosperm cellularization, which provide new insights into seed morphogenesis.

### Cell wall formation and modification during embryo differentiation and endosperm cellularization

It has been reported that the appearance of endosperm cell wall in the micropyle zone of seeds indicates that endosperm development transforms from the syncytial endosperm to cellularization stages (Otegui and Staehelin, 2000). Cell walls in plants contain various polysaccharides (cellulose, hemicellulose, and pectins), glycoproteins, and aromatic or aliphatic compounds, which form the mechanically strong and extensile network (Caffall and Mohnen, 2009). Cell walls provide mechanical strength for cells and fix the cell shape, which requires the modification and remodeling of the cell wall. The modification gene *XTH31*, coding a xyloglucan endotransglycosylase/hydrolase, is expressed in endosperm of *Arabidopsis* and can reinforce their cell walls during seed germination (Endo et al., 2012). ZHOUPI is an endosperm-specific gene in *Arabidopsis* and affects embryo and endosperm development by regulating the expression of pectin-modifying enzymes. In endosperm cell wall of *zou* mutant, (1-5)-α-L-arabinan epitopes immunized with the LM6 antibody were abundant, its endosperm cells are more robust than WT, and the cell wall fail to breakdown, resulting in the abnormal size of embryo (Fourquin et al., 2016). The focus of investigation of seed cell walls is mainly the roles of cell wall during seed germination; however, up to date, the transcriptional regulation of cell wall formation during endosperm cellularization is still unclear.

AGPs and FLAs are important proteoglycans that form covalent bonds with both hemicellulose and pectin in cell walls. In rice, AGP protein is widely distribution in tapetum cells, pollen mother cells, and mature pollens (Ma et al., 2019). In *FLA3* RNAi transgenic plants of *Arabidopsis*, pollen grains are shrunken and wrinkled, and cellulose abnormal distribution in pollen walls to lead to reduced male fertility (Li et al., 2010). APAP1 (ARABINOXYLAN PECTIN ARABINOGALACTAN PROTEIN1) encodes an AGP protein, and contributes to covalent linkages between cell wall proteins, pectins, and xylans. In *apap1*, the covalent linkages of pectin and xylan in cell walls of stem are less tightly (Tan et al., 2013). In our study, cell wall organization/biogenesis and cell wall terms were significantly enriched in embryo differentiation and endosperm cellularization of 3-5 DAP WT (**Supplementary Figure 2** and **Supplementary Table 10**). These DEGs were mainly annotated to genes related to proteins of cell walls (*AGP* and *FLA*) and its modification (**Supplementary Table 11**). Among them, 27 DEGs annotated to *pectin esterase*/*pectin methylesterases* (*PME*) showed lower expression levels in *naa15* (**Figure 7C**). This indicated that these DEGs might be involved in the formation of the cell wall during early seed development.

Meanwhile, through analyzing the distribution of esterified pectin during embryo and endosperm development, the immunolabeled results showed that low-esterification pectin of endosperm cell wall gradually decreased by using JIM5 antibody; in contrast, by using JIM7 antibody, the high-esterification pectin was increased in 4 and 5W (**Figures 8K, L, R**). Thus, we proposed that decreased low-esterification pectin and increased high-esterification pectin in cell walls might be largely related to endosperm cellularization, which increased the rigidity and stiffness of cell wall. In the endosperm cell walls of *naa15*, JIM5 and JIM7 signals showed lower fluorescence intensities than in WT (**Figures 8L, R**), but the JIM7 signals of embryos in WT and *naa15* had no significant differences (**Figure 8Q**). Based on the results, we speculated that the esterification feature change of the endosperm cell wall in *naa15* might be one of the causes of the abnormal endosperm. We hypothesized that the defective embryo and endosperm of the *naa15* mutant might be due to impairing the cell characteristics, which in turn affected embryo differentiation and endosperm cellularization. It has been reported that the embryo morphology and mucilage extrusion of seeds in *Arabidopsis hms-1* mutant were altered owing to methyl esterification degree of cell walls (Levesque-Tremblay et al., 2015). Hence, cell wall formation and modification were vital for embryo and endosperm during early seed development.

### Starch and sucrose metabolism and photosynthesis-related DEGs in early embryo and endosperm development

In GO and KEGG enriched analysis of these DEGs, starch and sucrose metabolism pathways were enriched in 3-5W (**Figures 3B-D**). Sucrose is imported into developing embryo by sucrose transporters (suc/sut) and sucrose exporter proteins (SWEET). The *sweet11;12;15* triple mutant in *Arabidopsis* disrupts sugar transport, resulting in delayed embryo development (Chen et al., 2015). In our research, the transcriptome levels of *SWEET5*, *SWEET11*, and *SWEET15* in 3-5W were upregulated, and sucrose metabolism genes such as *AT1G19450*, *SFP1*, and *SPS3* were also upregulated. However, their roles in seed development remain unclear. The starch and sucrose pathway was also significantly enriched using KEGG analysis in *naa15* compared with WT (**Figures 4E–I**). The heatmap of sucrose synthesis and sucrose transporter genes showed that *SUS1*, *SUS4*, *SUC1*, *SWT3*, *SWT4*, *SWT7*, and *SWT9* were significantly down-regulated in *naa15* compared with WT. Taken together, these results implied that sucrose metabolism may provide nutrients and energy for promoting normal cell growth of early seed development. However, the role of *NAA15* in sucrose metabolism remained unclear and needed to be further investigated.

From globular-embryo stage, the organ differentiation of embryo begins to occur, and the seed is transformed from the heterotrophic stage to the autotrophic stage. In embryonic cells, proplastids are converted into chloroplasts, accompanied by the biosynthesis of chlorophyll (Tejos et al., 2010). Therefore, lots of gene expressions related to photosynthesis are regulated during embryo differentiation and endosperm cellularization. Transcriptome GO analysis shows that photosynthesis term is significantly enriched in soybean seeds at globular, heart, and cotyledon stages, implying that photosynthesis might play important role in the embryo development (Sun et al., 2020). In many homozygous mutants, the abnormal development of chloroplasts leads to albino seeds and embryonic lethality. EMB1990 and EMB2726 are plastid-localized proteins, and the chloroplasts of *EMB1990* and *EMB2726* knockout mutants are severely impaired, resulting in defective embryos (Chen et al., 2018b; Li et al., 2021). It remains poorly understood what are intriguing biological functions of embryonic chloroplast in *Arabidopsis*.

In our transcriptome data, KEGG and GO analyses showed that many DEGs of 4W vs 5W group were enriched in photosynthesis term and chloroplast components (apoplast, thylakoid, and photosynthetic membrane) (**Supplementary Figures 4B–D**, **6**). Interestingly, six *photosystem I-antenna* (*LHCI*) genes and 12 *chlorophyll a/b binding protein complex Ⅱ* genes exhibited up-regulated expression trends at 3-5W, but the photosynthetic genes were dramatically downregulated in *naa15* (**Supplementary Figure 4B**), indicating that embryonic photosystem of the *naa15* mutant failed to function, which might be the reason for the white aborted seeds of *naa15*. However, further studies are needed to investigate the relationship between *NAA15* and genes related to photosystems.

### Phytohormone-related DEGs during embryo differentiation and endosperm cellularization

Plant hormones are crucial signaling molecules in seed development. It has been reported that auxin is involved in the communication between the endosperm, embryo, and seed coat, and proper embryo development requires the transportation of auxin between endosperm and integument (Batista et al., 2019). In the *agl62* mutant of *Arabidopsis*, the export of auxin is impaired, causing auxin accumulation in the endosperm, and cellularization prematurely occurs (Kang et al., 2008; Figueiredo et al., 2016). In the *auxin response factor 2* (*arf2*) mutant of *Arabidopsis*, extra cell proliferation occurs in the seed integuments, leading to enlarged seed coats and enhanced seed mass (Schruff et al., 2006). Additionally, it is well documented that auxin has an immediate negative control on cytokinin levels by inhibiting its biosynthesis (Nordström et al., 2004), and the ratio of auxin and cytokinin is critical for tissue proliferation and differentiation (Day et al., 2008). The cytokinin has important roles in root vascular patterning, and the septuple mutant *log1 log2 log3 log4 log5 log7 log8* shows severe embryonic vascular tissue development and patterning defects (De Rybel et al., 2014).

In our transcriptome data, the GO and KEGG analyses showed that the signal transduction pathways related to phytohormones were enriched in 3-5W (**Figure 3** and **Supplementary Table 10**), involving in auxin, cytokinin, and abscisic acid (**Supplementary Table 11**). In *naa15*, DEGs related to auxin response factor (ARF), auxin-responsive protein (IAA), and auxin efflux proteins were significantly down-regulated in *naa15* (**Figures 5A-C**). Hence, the auxin response and transport in *naa15* was disrupted owing to deletion of *NAA15*. In addition, the genes related to several cytokinin dehydrogenases and cytokinin-activating enzymes were also down-regulated in *naa15* (**Figure 5D**). It has been reported that cytokinin signaling is crucial during syncytial endosperm development (Day et al., 2008), and the homeostasis of auxin and cytokinin is essential for seed formation. Enhancing auxin or reducing cytokinin can disrupt embryo development (Zhang et al., 2017). Based on our transcriptional data, DEG-related hormones might be useful for investigating the roles of hormone regulation in these processes. However, further investigation into the relationship between *NAA15* and phytohormones (auxin and cytokinin) were required.

### Transcription factors in early embryo and endosperm development

The spatiotemporal expression of different genes is an important foundation during embryo and endosperm development and is regulated by their transcription factors (TFs). In early seed development of maize, transcriptome analysis shows that 110 TFs display high temporal specificity expression, and that *MYB131*, *MYB16*, and *BZIP109* might be critical for endosperm cellularization (Yi et al., 2019). The WOX, HD-Zip, ARF, and CUC families of the transcription factors have been reported to be crucial in the establishment of embryonic patterning (Verma et al., 2022). WOX2 and WOX8 are co-expressed in the zygote of *Arabidopsis*, and WOX2 expression in the apical region is activated by WOX8 and WOX9 to regulate proper embryo development (Breuninger et al., 2008). Besides, the *rev phb* (HD-Zip TF) double mutant displays single or radially symmetric cotyledon embryo patterning defects (Prigge et al., 2005). The WRKY transcription factor *MINISEED3* (*MINI3*) is expressed in both endosperm and globular embryos; hence, the endosperm proliferation in *mini3* mutant is retarded, leading to the reduced size of seeds (Luo et al., 2005).

In our research, the transcription regulator activity term was significantly enriched in 3-5W (**Supplementary Figure 3A**), but genes related to embryonic differentiation, such as *WOXs* (*WOX1*, *WOX2*, *WOX5*, *WOX8*), CUCs (*CUC2*, *CUC3*), and HD-Zip III (*ATHB8*, *REV*), exhibited downregulated expression in *naa15* (**Figure 6** and **Supplementary Table 15**). N-terminal acetylation of cellular proteins affects protein interactions, subcellular targeting, protein folding, and protein degradation (Scott et al., 2011; Yang et al., 2013). However, up to date, no correlations between *NAA15* and these genes were reported. We proposed that *NAA15* mutation in *Arabidopsis* led to NatA inactivation, which might cause some protein misfolding and excessive accumulation, resulting in abnormal expressions of these DEGs in *naa15* seeds. Additionally, 35 *MYB*, 37 *bHLH*, 30 *WYKY*, and 39 *ERFs* from our transcriptome data showed abnormal expressions in *naa15* (**Supplementary Table 14**). Among these transcription factors, except for *MYB56*, *bHLH95*, *WRKY10*, and *WRKY2*, which have been reported for their roles in embryo and endosperm (Ueda et al., 2011; Zhang et al., 2013; MacGregor and Zhang., 2019), there were no reports on other transcription factors. The results implied that the transcription factors might have important functions in embryo and endosperm development and could serve as potential regulators for seed morphogenesis.

## Data availability statement

The datasets presented in this study can be found in online repositories. The names of the repository/repositories and accession number(s) can be found below: NCBI Bioproject accession number is PRJNA856854.

## Author contributions

JZ designed the program of studies and experiments, guided the entire process of the study, and revised the manuscript. CL finished the material collection, carried out most of the experiments, analyzed the data, and wrote the paper. FH performed qPCR analyses. HC performed endosperm autofluorescence assay. All authors read and approved the content of the paper. All authors contributed to the article and approved the submitted version.

## Funding

This work was supported by the National Natural Science Foundation of China (31870303, 32170337).

## Conflict of interest

The authors declare that the research was conducted in the absence of any commercial or financial relationships that could be construed as a potential conflict of interest.

## Publisher’s note

All claims expressed in this article are solely those of the authors and do not necessarily represent those of their affiliated organizations, or those of the publisher, the editors and the reviewers. Any product that may be evaluated in this article, or claim that may be made by its manufacturer, is not guaranteed or endorsed by the publisher.

## Supplementary material

Supplementary Figure 1 Seed characteristics and genotype analysis of wild type and *naa15*/+ mutants. **(A)** Seed phenotypes in siliques of the wild type (WT) and *naa15*/+. The homozygous white aborted *naa15* seeds are labeled by white arrows. Bar = 1mm. **(B)** Genotypic analysis of WT and *naa15*/+ mutants. FP, forward primer; RP, reverse primer.

Supplementary Figure 2 Global analysis of the RNA-seq profile. **(A)** Violin map of gene expression distribution. The vertical axis meant the value change of the gene expression using log_10_ (FPKM). The horizontal axis represented eighteen biological samples. **(B)** Principal component analysis (PCA) of eighteen biological samples. **(C)** Pearson-correlation coefficients showed the repeatability of the samples.

Supplementary Figure 3 Gene Ontology (GO) enriched terms of DEGs in 4W vs 3W and 5W vs 4W of *Arabidopsis*. **(A,B)** GO enrichment analysis of up-regulated DEGs in 4W vs 3W and 5W vs 4W W, respectively. **(C,D)** GO enrichment analysis of down-regulated DEGs in 4W vs 3W and 5W vs 4W, respectively.

Supplementary Figure 4 Heatmaps of DEGs related to sucrose and photosynthesis in WT and *naa15* of *Arabidopsis*. **(A)** Heatmap of DEGs related to sucrose synthesis and transport in WT and *naa15*. **(B)** Heatmap of DEGs related to photosynthesis in WT and *naa15*.

Supplementary Figure 5 Gene Ontology (GO) enriched terms of down-regulated DEGs from comparisons between WT and *naa15* in 5M vs 3W, 6M vs 4W, and 7M vs 5W of *Arabidopsis*.

Supplementary Figure 6 Gene Ontology (GO) enriched terms of up-regulated DEGs from comparisons between WT and *naa15* in 5M vs 3W, 6M vs 4W, and 7M vs 5W of *Arabidopsis*.
