# Supplementary figures and images for: Transcriptome characteristics during cell wall formation of endosperm cellularization and embryo differentiation in Arabidopsis
Source: Front Plant Sci. 2022 Oct 3;13:998664. doi: 10.3389/fpls.2022.998664 (PMC9575994; doi:10.3389/fpls.2022.998664)

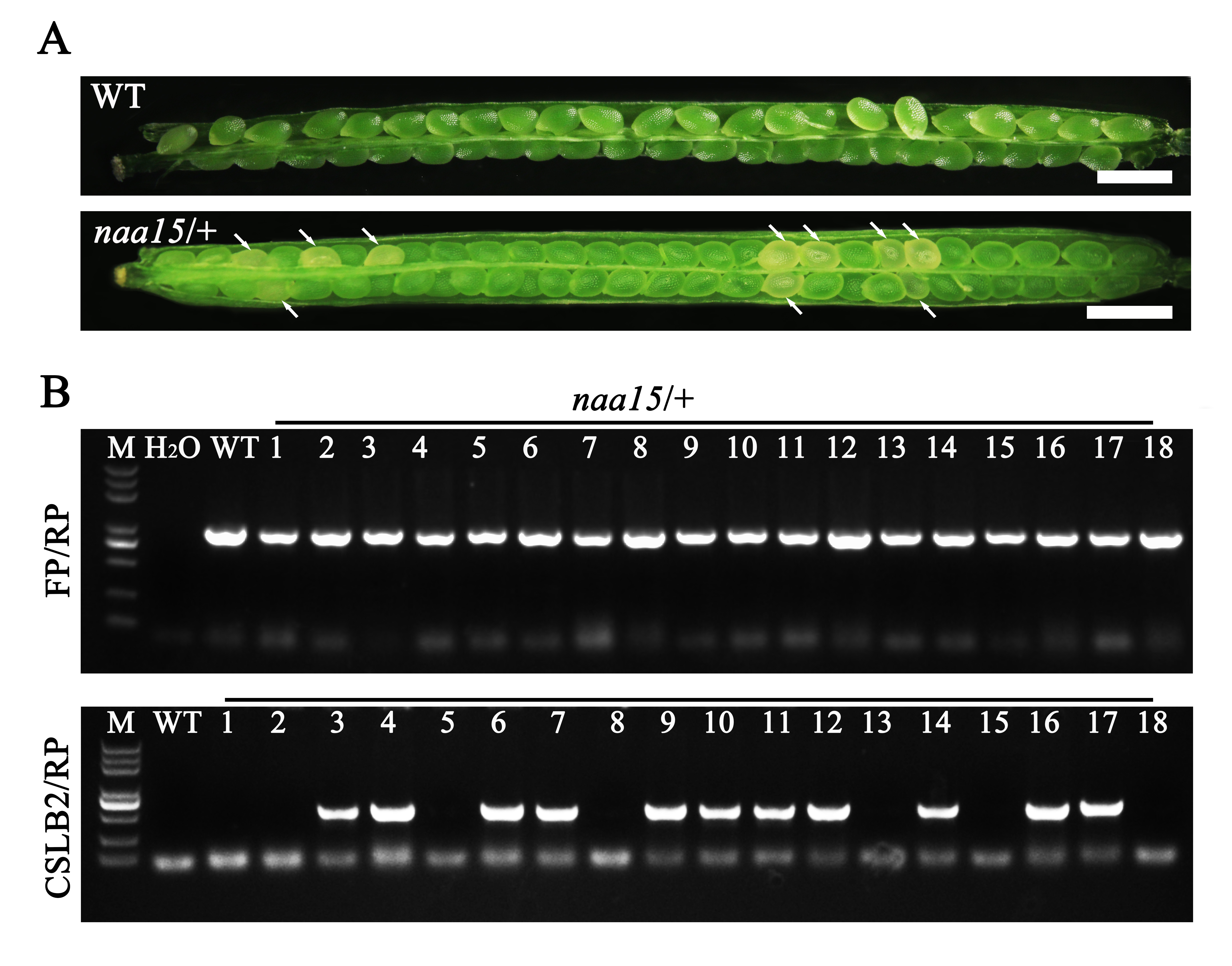

Supplement: Supplementary Figure 1 — Seed characteristics and genotype analysis of wild type and naa15/+ mutants. (A) Seed phenotypes in siliques of the wild type (WT) and naa15/+. The homozygous white aborted naa15 seeds are labeled by white arrows. Bar = 1mm. (B) Genotypic analysis of WT and naa15/+ mutants. FP, forward primer; RP, reverse primer. [file Image_1.jpeg]

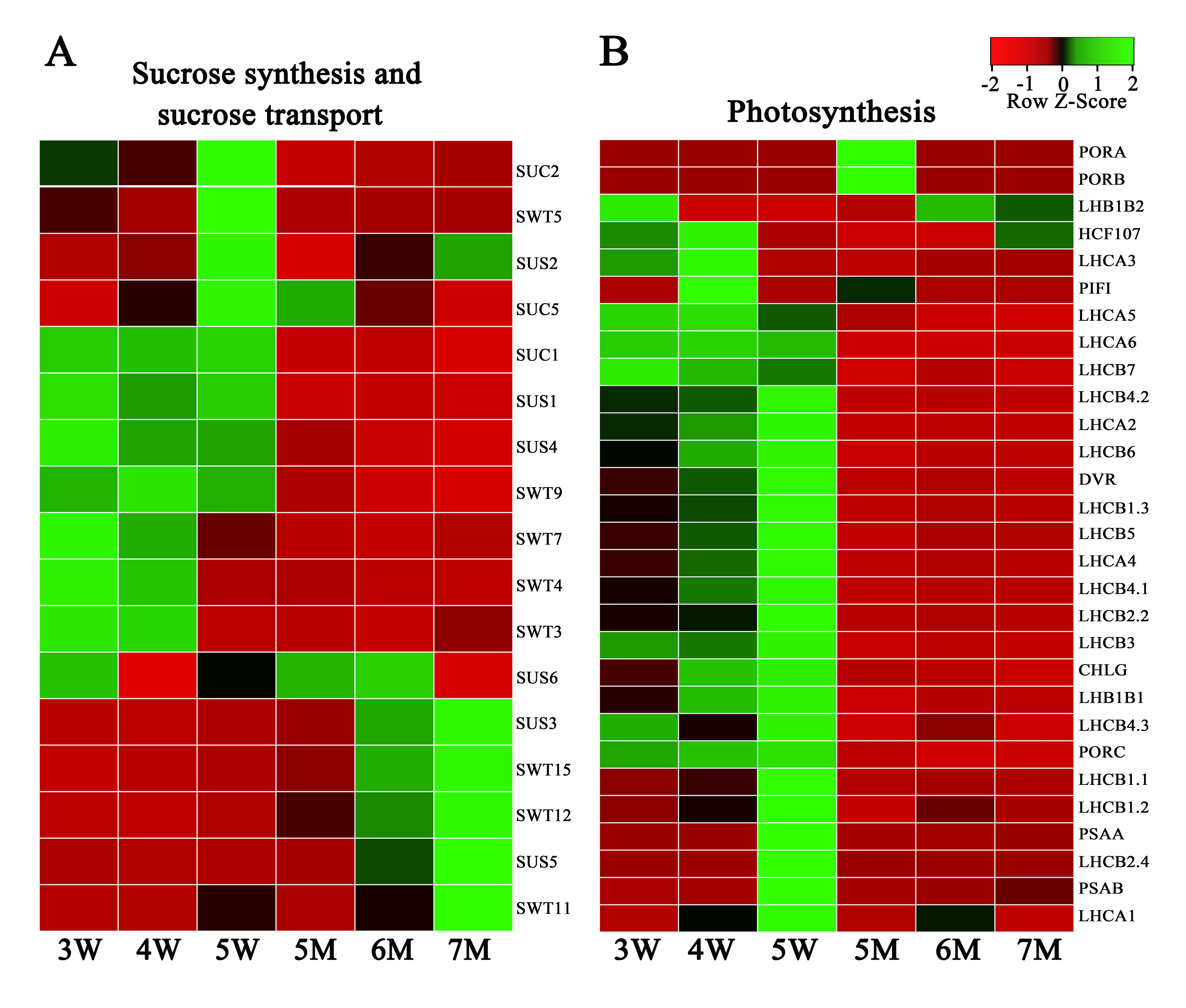

Supplement: Supplementary Figure 4 — Heatmaps of DEGs related to sucrose and photosynthesis in WT and naa15 of Arabidopsis. (A) Heatmap of DEGs related to sucrose synthesis and transport in WT and naa15. (B) Heatmap of DEGs related to photosynthesis in WT and naa15. [file Image_4.jpeg]
